# Prevalence and multi-locus genotyping of *Giardia duodenalis* in rabbits from Shaanxi province in northwestern China

**DOI:** 10.1051/parasite/2021052

**Published:** 2021-06-25

**Authors:** Huan Tang, Yonggang Ye, Runmin Kang, Jifeng Yu, Ye Cao

**Affiliations:** 1 Animal Breeding and Genetics Key Laboratory of Sichuan Province, Sichuan Animal Science Academy Chengdu 610066 PR China; 2 College of Veterinary Medicine, Northwest A&F University Yangling Shaanxi 712100 PR China; 3 Chongqing Three Gorges Vocational College Chongqing 404155 PR China

**Keywords:** *Giardia duodenalis*, Multi-locus genotyping, Prevalence, Rabbit, Shaanxi Province

## Abstract

*Giardia duodenalis* is an important parasite with veterinary and public health significance worldwide. The presence and zoonotic assemblages of *G. duodenalis* have previously been reported in rabbits. In this study, to understand the infection status of *G. duodenalis* in rabbits from Shaanxi province, a total of 537 fecal samples were collected from two breeds of rabbits in four age groups (<30 days, 31–90 days, 91–200 days and >200 days) from four geographical origins (Fengxiang, Yangling, Tongchuan, and Shanyang). The presence of *G. duodenalis* in these samples was assessed using molecular assays based on beta-giardin (*bg*). The glutamate dehydrogenase (*gdh*) and triosephosphate isomerase (*tpi*) loci were then amplified in the *bg*-positive samples for multi-locus genotype (MLG) analysis. The total prevalence of *G. duodenalis* in these rabbits was 3.54% (19/537). *Giardia duodenalis* infection was found in both breeds of rabbits, and in all farms and age groups, but with no statistically significant differences related to these factors (*p* > 0.05). Two assemblages, including B and E, were identified, with the former the predominant assemblage detected in both breeds, and in all age groups and farms. Sequence analysis revealed 2 (named as rbg1-2), 1 (named as rtpi1), and 2 (named as rgdh1-2) haplotypes at the gene loci of *bg*, *tpi,* and *gdh*, respectively, forming a multilocus genotype (MLG) of assemblage B (rbg1, rtpi1, and rgdh1). These findings reveal the significant zoonotic potential and genetic diversity of *G. duodenalis* in rabbits in Shaanxi Province, PR China.

## Abstract (fr)

*Giardia duodenalis* est un parasite de grande importance vétérinaire et en santé publique dans le monde entier. La présence et les assemblages zoonotiques de *G. duodenalis* ont déjà été rapportés chez le lapin. Dans cette étude, pour comprendre le statut infectieux de *G. duodenalis* chez les lapins de la province du Shaanxi, un total de 537 échantillons fécaux ont été prélevés sur deux races de lapins dans quatre groupes d’âge (<30 jours, 31–90 jours, 91–200 jours et >200 jours) de quatre origines géographiques (Fengxiang, Yangling, Tongchuan, Shanyang). La présence de *G. duodenalis* dans ces échantillons a été évaluée à l’aide de tests moléculaires basés sur la bêta-giardine (*bg*). Les loci de la glutamate déshydrogénase (*gdh*) et de la triosephosphate isomérase (*tpi*) ont ensuite été amplifiés dans les échantillons *bg*-positifs pour l’analyse des génotypes multilocus (MLG). La prévalence totale de *G. duodenalis* chez ces lapins était de 3,54 % (19/537). L’infection à *Giardia duodenalis* a été trouvée chez les deux races de lapins et dans tous les élevages et groupes d’âge, mais sans différence statistiquement significative liée à ces facteurs (*p* > 0,05). Deux assemblages, dont B et E, ont été identifiés, le premier étant l’assemblage prédominant détecté dans les deux races, et dans tous les groupes d’âge et élevages. L’analyse des séquences a révélé des haplotypes, 2 (nommés rbg1-2), 1 (nommé rtpi1) et 2 (nommés rgdh1-2) aux loci des gènes de *bg*, *tpi* et *gdh*, respectivement, formant un génotype multilocus (MLG) de l’assemblage B (rbg1, rtpi1 et rgdh1). Ces résultats ont révélé l’important potentiel zoonotique et la diversité génétique de *G. duodenalis* chez les lapins de la province chinoise du Shaanxi.

## Introduction

*Giardia duodenalis* is an important intestinal parasite of humans and more than 40 animal species, making it the 11th most important foodborne parasite globally according to the Food and Agriculture Organization of the United Nations (FAO)/World Health Organization (WHO) [8, 18, 20, 59]. Giardiasis, caused by *G. duodenalis*, is one of most common diarrheal diseases in animals and humans, and is responsible for approximately 280 million human diarrheal cases reported annually worldwide [10, 15, 59]. The most common symptoms in infected individuals are foul-smelling diarrhea, greasy stools, flatulence, and bloating, and death can occur in children five years of age or younger in low-income countries [4, 6, 13, 23, 28, 35, 36, 63]. Although asymptomatic infection has been seen in most hosts, especially animals, viable cysts of *G. duodenalis* excreted from these individuals can be potential transmission sources for other animals and humans through waterborne and foodborne chains [14, 26, 59].

Molecular characterization of *G. duodenalis* revealed a species complex for this parasite, and eight valid assemblages (namely A–H) have been identified [16, 44, 46–48]. Zoonotic assemblages A and B have been found in both humans and animals, and some animal-adapted assemblages (C, D, E, and F) have also been detected in humans, suggesting possible transmission between humans and animals [20, 53, 60, 64, 69].

Rabbits are an important economically farmed animal, and it is also increasingly bred as a family pet [7, 12, 54]. However, rabbits can carry zoonotic pathogens, including *G. duodenalis* [56, 65]. The infection rate of *G. duodenalis* ranges from 1.90% to 72.30% [3, 31, 41, 42, 50, 56, 70, 71]. Three assemblages have been identified in rabbits, A, B and E, and all are potential sources of infection for humans [3, 27, 29, 41, 55, 70, 71], suggesting a potential role of rabbits in the transmission of *G. duodenalis*. China is the biggest rabbit meat producer around the world [38], with 849,150 tons [40]. In Shaanxi Province, rabbit breeding is increasing in prevalence, with local farms and larger agricultural companies. However, to date there have been only six reports on *G. duodenalis* infection from six Chinese provinces (Xinjiang, Heilongjiang, Liaoning, Henan, Jilin, and Shandong) [30, 41, 42, 56, 70, 71]. In this study, the prevalence and genetic diversity of *G. duodenalis* in farmed rabbits from Shaanxi province were investigated using a multilocus genotyping tool [11, 43, 67].

## Materials and methods

### Ethics statement

The fecal samples were collected with the farm owner’s permission. All procedures of our study were reviewed and approved by the Research Ethics Committee of Northwest A&F University, Yangling, Shaanxi.

### Sample collection

**Figure 1:**
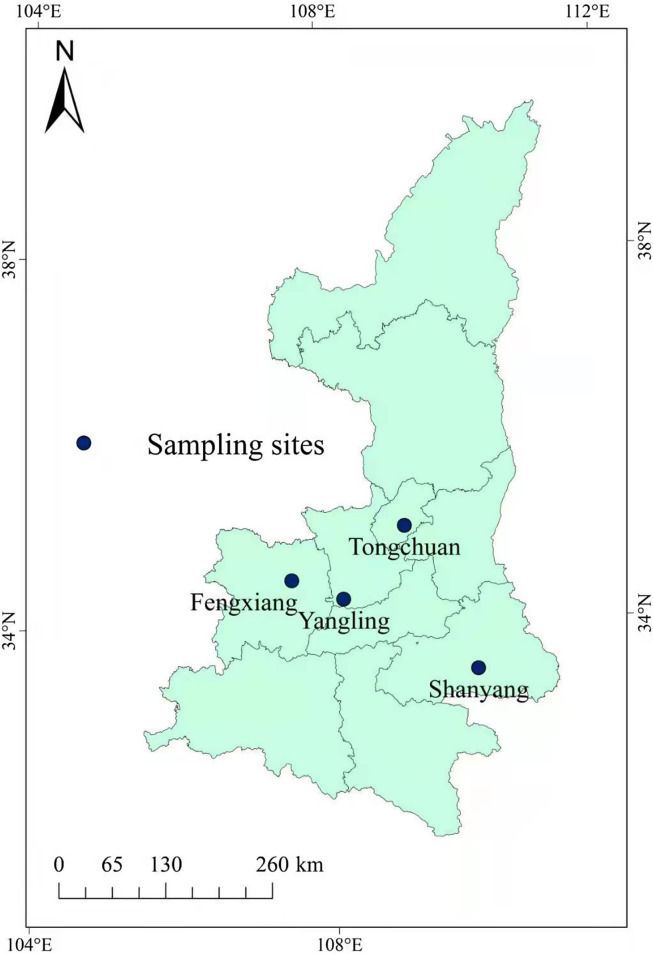
Sampling sites in this study.

A total of 537 fecal samples were collected from family farms or large-scale rabbit farms that breed approximately 1000–5000 rabbits. The selected farms are located in four geographical regions (Fengxiang, Yangling, Tongchuan, and Shanyang) in Shaanxi Province (105°29′–111°15′ E, 31°42′–39°35′ N). Samples were collected in July 2017 and March 2018 (Fig. 1). All fecal samples were randomly collected from a single cage containing between one and five rabbits of the same age (<30 days, 31–90 days, 91–200 days or >200 days). All feces collected from a cage were considered a single sample. Each sample was placed into a clean plastic bag and marked with the date, age, and geographical origin. All fecal samples were quickly transported to the laboratory with ice packs, preserved in 2.5% potassium dichromate, and stored at 4 °C for further study.

### Genomic DNA extraction

About 200 mg of each fecal sample were washed with distilled water, as described previously [7, 58]. Next, the genomic DNA was extracted from each sample using a commercial kit, according to the manufacturer’s instructions (E.Z.N.A^®^ stool DNA kit, Omega Bio-Tek Inc., Norcross, GA, USA). All genomic DNA samples were stored at –20 °C for further analysis.

### Nested PCR amplification and agarose gel electrophoresis

To determine the prevalence of *G. duodenalis* in rabbits in Shaanxi province, all genomic DNA extracted from fecal samples was subjected to nested PCR targeting the *beta giardin* (*bg*) gene, as reported previously [8, 39]. Next, all *bg*-positive samples were subjected to nested PCR reactions targeting the gene loci of *triose phosphate isomerase* (*tpi*) and or *glutamate dehydrogenase* (*gdh*) [39, 64]. Each PCR reaction included 15.375 μL dd H_2_O, 2.5 µL 10× Ex Taq Buffer (Mg^2+^ free) (Takara Bio Inc., Dalian, PR China), 2 µL MgCl_2_ (25 mmol/L) (Takara Bio Inc., Dalian, PR China), 2 µL dNTP mixture (2.5 mmol/L) (Takara Bio Inc., Dalian, PR China), 0.125 µL TaKaRa Ex Taq, 1 µL forward primer, 1 µL reverse primer, and 1 µL DNA samples. All PCR products were detected by 1% agarose gel electrophoresis with ethidium bromide staining and visualized using a UV transilluminator (Beijing Sagecreation Technology Co., Ltd, Beijing, PR China).

### Sequencing and sequence analysis

All positive PCR amplicons were sent to Sangon Biotech Co., Ltd, Shanghai, China for direct sequencing from both directions using the inner primers of nested PCRs. The obtained sequences were aligned with reference sequences downloaded from GenBank within the National Center for Biotechnology Information (NCBI) (KJ888980 for assemblage B at *bg* loci, KY769090 for assemblage E at *bg* loci, EU594666 for assemblage B at *gdh* loci, and HQ666894 for assemblage B at *tpi* loci) (Table 2) using ClustalX 1.83. Alignments were then corrected manually. Each corrected sequence was aligned using the Basic Local Alignment Search Tool (BLAST) within NCBI to determine assemblages of *G. duodenalis*, and all corrected sequences of each gene locus were aligned with reference sequences downloaded from GenBank to investigate the genetic diversities of *G. duodenalis* isolates.

### Statistical analysis

Differences in prevalence of *G. duodenalis* in rabbits from different geographical origins and age groups were analyzed using chi-squared (*χ*^*2*^) tests within SPSS 19.0 for Windows (SPAA Inc., Chicago, IL, USA). The statistical differences were considered significant when *p* < 0.05.

### Nucleotide sequence accession numbers

The 19 nucleotide sequences were compared with each other and identical sequences were grouped into one. Finally, five different sequences were obtained and deposited in GenBank with the following accession numbers: MN123235 and MN123236 for the *bg* gene, MN123234 for the *tpi* gene, and MN123237 and MN123238 for the *gdh* gene.

## Results

### Prevalence of *G. duodenalis*

A total of 537 rabbits fecal samples from four age groups of two breeds (Rex and IRA rabbits) from five farms in four counties/cities were examined. A total of 19 rabbit fecal samples were identified as positive for *G. duodenalis* infection based on nested-PCR targeting the *bg* gene, with a total prevalence of 3.54% (19/537). *G. duodenalis* was detected in both Rex (3.68%) and IRA rabbits (3.49%), with a slightly higher prevalence found in Rex rabbits (Table 1). All examined farms were positive for *G. duodenalis* infection, with prevalence ranging from 1.45% (3/207) (Farm 4 in Shanyang) to 8.43% (7/83) (Farm 3 in Tongchuan) (Table 1). *Giardia duodenalis* was detected in rabbits of all age groups, with the highest detection (10.53%) in animals less than 30 days old, and lowest detection (2.58%) in animals more than 200 days old (Table 1). However, no significant differences in prevalence were detected among rabbits of different farms (χ^2^ = 7.737, df = 4, *p* > 0.05), breeds (χ^2^ = 0.028, df = 1, *p* > 0.05) or age groups (χ^2^ = 3.970, df = 3, *p* > 0.05).

**Table 1:** Prevalence of *Giardia duodenalis* infection in rabbits in Shaanxi Province, northwestern China.

Variable	Category	No. positive (%)	Target locus (no. positive)		
			*bg*	*gdh*	*tpi*
Breed	Rex rabbit	3.68 (5/136)	5	4	2
	IRA rabbit	3.49 (14/401)	14	1	6
	Total	3.54 (19/537)	19	5	8
Geographical origin	Fengxiang	3.68 (5/136)	5	4	4
	Yangling	4.26 (2/47)	2	0	1
	Tongchuan	8.43 (7/83)	7	1	2
	Shanyang	1.85 (5/271)	5	0	3
	Total	3.54 (19/537)	19	5	8
Farm	Farm1	3.68 (5/136)	5	4	2
	Farm2	4.26 (2/47)	2	0	1
	Farm3	8.43 (7/83)	7	1	2
	Farm4	1.45 (3/207)	3	0	2
	Farm5	3.13 (2/64)	2	0	1
	Total	3.54 (19/537)	19	5	8
Age (days)	<30	10.53 (2/19)	2	2	2
	31–90	4.35 (5/115)	5	0	3
	91–200	3.79 (5/132)	5	2	1
	>200	2.58 (7/271)	7	1	2
	Total	3.54 (19/537)	19	5	8
Assemblages	B	18	18	5	8
	E	1	1	–	–
	Total	19	19	5	8
MLG type	MLG B	3	–	–	–
	Total	3	–	–	–

### Detecting of two assemblages

Assemblage B and assemblage E were detected in this study. Assemblage B was identified in 18 samples (Table 2). It was detected in both Rex (3.68%) and IRA rabbits (3.49%), as well as on all farms and in age groups (Table 1). Assemblage E was identified in only one sample, which was collected in Rex rabbits from Fengxiang.

**Table 2:** Intra-assemblage substitutions in *bg*, *tpi* and *gdh* sequences within assemblage B and assemblage E.

Subtypes (the number of isolates)	Nucleotide positions and substitutions					GenBank IDs
	34	53	113	318	370	
*bg*						
B (Ref. sequence)	A	C	C	G	A	KJ888980
rbg1 (*n* = 18)	A	C	C	G	A	MN123235
E (Ref. sequence)	A	G	A	A	C	KY769090
rbg2 (*n* = 1)	A	G	A	A	T	MN123236
*gdh*						
B (Ref. sequence)	C	A	C	T	C	EU594666
rgdh1 (*n* = 4)	C	A	C	C	C	MN123237
rgdh2 (*n* = 1)	C	A	T	T	C	MN123238
*tpi*						
B (Ref. sequence)	T	T	T	A	T	HQ666894
rtpi1 (*n* = 8)	T	T	T	A	T	MN123234

### Genetic variability examination

At the *bg* gene locus, two haplotypes (named rbg1, rbg2) (Table 2) were identified. BLAST search showed that the sequence of haplotype rbg1 was identical to that of *G. duodenalis* assemblage B isolates from Lemur catta (KJ888980) [32], while haplotype rbg2 exhibited high sequence identity to *G. duodenalis* assemblage E isolates from dairy cattle in China (KY769090) [66], with a nucleotide transition (C-T) at position 370 (Table 2). All 19 *bg*-positive samples were analyzed by nested PCRs targeting the gene loci of *tpi* and *gdh*, with eight and five samples, respectively, successfully amplified for these loci. Sequence analysis showed one (named as rtpi1) and two (named rgdh1, rgdh2) (Table 2) haplotypes at the gene loci of *tpi* and *gdh,* respectively. The sequence of haplotype rtpi1 exhibited 100% identity to assemblage B isolates from rabbits in China (HQ666894) [70]. The *gdh* haplotypes rgdh1 and rgdh2 have one base difference with a human reference sequence from Cuba (EU594666) (Table 2) [52].

## Discussion

*Giardia duodenalis* has been widely reported in animals worldwide, with prevalence of 1.6–91.33% [17, 19, 20, 24, 25, 68]. In this study, a total of 19 rabbit fecal samples were identified as positive for *G. duodenalis* infection by nested-PCR targeting the *bg* gene, for a total prevalence of 3.54% (19/537). This is in the range of prevalence for *G. duodenalis* infection, but lower than most previous studies, e.g. 8.40% in Henan [56], 7.41% in Heilongjiang [70], 7.60% in Europe [51], 13.79% in Jilin [30], 11.20% in Shandong [41], and 72.30% in Nigeria [3]. However, it was higher than that detected in Liaoning (1.47%) [30] and Xinjiang (1.90%) [71]. The different reports of *G. duodenalis* prevalence in rabbits in different studies may reflect the different examination methods used. For example, a study in European countries used coproantigen ELISA to investigate the prevalence [51], while previous studies in Heilongjiang, Henan, Jilin, and Liaoning [30, 56, 70] used Lugol’s iodine staining with microscopic analysis. Microscopy analysis may underestimate prevalence, since low infection intensity may not be detected and expertise is required [50, 56, 57]. Measurements of the prevalence of *G. duodenalis* in rabbits from Henan (3.35%) [56] and Xinjiang (1.90%) [71] by PCR were both lower than the prevalence in this study. However, there may also be variation in *G. duodenalis.* A study from Nigeria [3] revealed a much higher prevalence (72.30%) than in Shaanxi, potentially due to differences in livestock rearing and feeding environments. The rabbits in Nigeria were fed freshly cut forage, while the rabbits in this study received pellet feed [3]. Additionally, the rabbits studied in Nigeria were raised in close proximity to other animal species (cattle, sheep, goats, pigs, and poultry), which may increase *G. duodenalis* infection. A previous study in Shandong used the same target gene as this study to detect prevalence, with higher prevalence (11.2%) than that detected here [41]. This difference may reflect the different breeds studied, as Long-haired and New Zealand white rabbits were studied in Shandong, but Rex and IRA rabbits were tested in Shaanxi [41]. Feeding practices may alter prevalence, with lower rates of infection in indoors rabbits (4.57%) than in outdoors rabbits (23.08%) in Shandong [41]. Overall, detection methods, sampling strategies, ecological and geographical environments, management practices, and husbandry modes can affect prevalence.

No significant differences in prevalence were detected among rabbits from different farms and different age groups in our study, suggesting that the infection of *G. duodenalis* in Shaanxi province may not be affected by these factors. A previous study conducted in Henan province, China assessed differences in prevalence among rabbits from different farms and age groups and found no significant differences (χ^2^ = 75.79, df = 8, *p* < 0.01) among rabbits from nine farms or in different age groups (χ^2^ = 5.69, df = 3, *p* > 0.05) [54]. Future work should measure a greater number of samples of various rabbit breeds from more geographical origins.

Three *G. duodenalis* assemblages, A, B and E, were previously reported in rabbits [30, 42, 56, 70, 71]. In this study, assemblage B was identified in 18 samples and prevalent in Rex (3.68%) and IRA rabbits (3.49%), and on all farms and age groups (Table 1). These findings were consistent with those from studies in Nigeria and in Henan, Xinjiang, Heilongjiang, Jilin, and Liaoning provinces, China [3, 30, 42, 56, 70, 71]. Of two common assemblages (A and B) in humans, genotype AI is considered zoonotic, genotype AII is mainly found in humans, and assemblage AIII is found exclusively in animals. For assemblage B, genotypes BIII and BIV are potentially zoonotic [9, 62]. The role of the genetic diversity of *G. duodenalis* and its clinical appearance is a controversial topic. Some reports showed that asymptomatic infection was associated with infection in children [2, 20, 29], but other reports associated clinical symptoms (being underweight, duodenal inflammation) with assemblage B, and asymptomatic individuals of *G. duodenalis* related to assemblage A [20, 36]. The differences may reflect genetic differences among the isolates of assemblages, the multiplication rate of parasites, the interplay with host factors, and the changes of transmission dynamics [20, 33, 37, 45, 49, 62]. Assemblage B has been detected in other animals, including beavers, cattle, dogs, horses, monkeys, muskrats, and sheep [8], and severe clinical symptoms were observed in lambs infected with *G. duodenalis* assemblage B, including malodorous and poorly formed feces and severe weight loss [5]. These findings suggest the importance of *G. duodenalis* assemblage B in both humans and animals. There is also evidence that assemblage E, a previously hoofed animal-specific assemblage, has zoonotic potential, since assemblage E has been detected in humans from some areas with poor conditions, such as Egypt and Brazil [1, 21]. These results suggested that it is important to be aware of the potential transmission of *G. duodenalis* from rabbits to humans and other animals in Shaanxi province. Future work should increase testing by rabbit farmers to confirm the prevalence and genotypes of *G. duodenalis*.

Genetic variability has been detected within *G. duodenalis* isolates from humans and animals [20, 27]. Assemblage B was the major genotype detected, and haplotype rbg1 obtained at the *bg* gene locus was identical to that in assemblage B isolates from rabbits, Lemur catta, and humans [54, 65]. Haplotype rbg2 has high sequence identity (99.76%) to assemblage E isolates from dairy cattle, Tibetan sheep, foals, Capra hircus, Ovis aries, Bos Taurus, and lambs [22, 24, 31, 34, 65]. Both B and E assemblages identified in this study have been reported in humans [20]. To further examine the genetic diversity of *G. duodenalis* isolates from rabbits in Shaanxi Province, eight and five samples were amplified at the gene loci of *tpi* and *gdh*, respectively. The haplotype rtpi1 was identified as assemblage B-IV identical to the isolates from rabbits in China [70, 71]. The *gdh* haplotype (rgdh1) was identical to *G. duodenalis* assemblage B isolates from humans in Zambia, Canada, Brazil, Poland, and water in Canada [55, 61]. Haplotypes rgdh1 and rgdh2 exhibited high sequence identity to sequences isolated from humans in Cuba [52]. Interestingly, three samples were successfully amplified at all three gene loci, forming a multilocus genotype (MLG) of assemblage B (rbg1, rtpi1, and rgdh1) (Table 3). Two were identified in rabbits aged < 30 days from Fengxiang, and one was detected in 31–90-day-old rabbits from Tongchuan.

**Table 3:** Multilocus characterization of *Giardia duodenalis* isolates based on the *bg*, *tpi* and *gdh* genes.

Isolate	Genotype or subtype			MLG type
	*bg*	*tpi*	*gdh*	
FxA2, FxA12, TcC11	rbg1	rtpi1	rgdh1	MLG B
FxC30	rbg1	–	rgdh2	–
FxE11	rbg1	–	rgdh1	–
Sh1A90, Sh1A97, Sh2C29, TcB17, TxA18	rbg1	rtpi1	–	–
Sh1B38, TcB8,TcB13, TcB14, TcC5, TcC29, Tx3, Tx18	rbg1	–	–	–
FxD8	rbg2	–	–	–

This study is the first report of *G. duodenalis* infection in rabbits from Shaanxi province, with a total prevalence of 3.54%. *Giardia duodenalis* was detected in both Rex and IRA rabbits, and detected on all farms and in all age groups (Table 1). Assemblages B and E were identified in these rabbits, with the zoonotic assemblage B predominant (18 isolates) (Table 2). Genetic diversity of assemblage B isolates in rabbits was also detected. Overall, these findings suggest zoonotic potential and genetic variation of *G. duodenalis* from rabbits in Shaanxi province, and provided the basis to implement control strategies of *G. duodenalis* in this province as well as other regions of the world.

## Conflict of interest

We declare that we do not have any commercial or associative interests that represent a conflict of interest in connection with the work submitted.
